# Learning multisensory cue integration: A computational model of crossmodal synaptic plasticity enables reliability-based cue weighting by capturing stimulus statistics

**DOI:** 10.3389/fncir.2022.921453

**Published:** 2022-08-08

**Authors:** Danish Shaikh

**Affiliations:** SDU Biorobotics, Maersk Mc-Kinney Moller Institute, University of Southern Denmark, Odense, Denmark

**Keywords:** multisensory cue integration, maximum-likelihood estimation, crossmodal learning, synaptic plasticity, biorobotics

## Abstract

The brain forms unified, coherent, and accurate percepts of events occurring in the environment by integrating information from multiple senses through the process of multisensory integration. The neural mechanisms underlying this process, its development and its maturation in a multisensory environment are yet to be properly understood. Numerous psychophysical studies suggest that the multisensory cue integration process follows the principle of Bayesian estimation, where the contributions of individual sensory modalities are proportional to the relative reliabilities of the different sensory stimuli. In this article I hypothesize that experience dependent crossmodal synaptic plasticity may be a plausible mechanism underlying development of multisensory cue integration. I test this hypothesis *via* a computational model that implements Bayesian multisensory cue integration using reliability-based cue weighting. The model uses crossmodal synaptic plasticity to capture stimulus statistics within synaptic weights that are adapted to reflect the relative reliabilities of the participating stimuli. The model is embodied in a simulated robotic agent that learns to localize an audio-visual target by integrating spatial location cues extracted from of auditory and visual sensory modalities. Results of multiple randomized target localization trials in simulation indicate that the model is able to learn modality-specific synaptic weights proportional to the relative reliabilities of the auditory and visual stimuli. The proposed model with learned synaptic weights is also compared with a maximum-likelihood estimation model for cue integration *via* regression analysis. Results indicate that the proposed model reflects maximum-likelihood estimation.

## 1. Introduction

Multisensory cue integration is a fundamental operation performed by the brain in the general process of multisensory integration. Cue integration is critical for spatial localization, which is crucial for the successful execution of higher level tasks such as navigation and object manipulation. Multisensory cue integration entails combining cues from different sensory modalities containing information about relevant events if the cues arise from the same perceptual source. Multisensory cue integration can enhance the unified percept of the event formed in the brain, relative to that formed from cues from a single sensory modality (Chen and Spence, 2017). For example, auditory and visual directional cues are typically integrated together in spatial localization tasks, to determine the spatial location of an audio-visual target.

Multimodal cues participating in the cue integration process can vary significantly in their respective reliabilities. For example, the visual sense is more accurate in the frontal space while the auditory sense is more accurate at the periphery (Odegaard et al., 2015), in the head-centered reference frame. For cue integration to generate the most reliable and unbiased estimate, i.e., one that exhibits minimum uncertainty or variance, individual sensory information should ideally be weighted in proportion to its relative reliability according to maximum-likelihood estimation (MLE) (Ernst and Banks, 2002).

The leading consensus in the scientific community posits that cue integration can be explained within the Bayesian framework. Behavioral studies suggest that sensory cue integration is Bayes-optimal or near optimal (Alais and Burr, 2004; Morgan et al., 2008; Fetsch et al., 2012). This process is probabilistic, in that cue reliability is taken into account, and Bayesian in the sense that prior information can be combined with available sensory information (Seilheimer et al., 2014). This suggests that the sensory cue reliability may be directly encoded in the brain.

### 1.1. Modeling multisensory cue integration and its development

Conventional models of multisensory cue integration, both at the single neuron level and at the population level, are based on Bayesian approaches. A review of models and processes of multisensory cue integration can be found in Seilheimer et al. (2014). Bayesian cue integration employs a weighted summation where cues are weighted by their corresponding reliabilities. Assuming that cues are generated from an underlying probability distribution, cue reliabilities are modeled *via* likelihood functions with multivariate Gaussian or Poisson distributions. Such weighted summation results in the cue integration being biased toward the more reliable cue. However, these models are typically applied to account for behavioral data from psychophysical experiments and do not shed light on the underlying neural mechanisms. Single neuron models of Bayesian approaches assume that the modality specific synaptic weights influencing the individual sensory cues represent cue reliabilities (Ohshiro et al., 2011). Neural population models assume that the likelihood of a sensory signal may be encoded in the combined neural activity of populations of neurons as demonstrated in simulation studies (Ma et al., 2006).

While there has been significant research in modeling multisensory cue integration, relatively few studies in the literature have reported on developmental models of multisensory cue integration. A neural network based population model of audio-visual integration that learned Bayesian cue combination from stimuli statistics has recently been reported (Ursino et al., 2017). More recently, I and colleagues developed a single neuron model that learned audio-visual cue integration in real time during spatial localization tasks. The model also exhibited neurophysiological phenomena such as multisensory depression and multisensory enhancement (Shaikh et al., 2019).

### 1.2. Neural correlates underlying multisensory cue integration and its development

Multimodal sensory signals have been reported to be processed in single granule cells in the cerebellum (Azizi and Woodward, 1990). Multisensory integration has been observed in these neurons *in vivo*, which exhibited enhanced action potentials (Ishikawa et al., 2015) in response to multimodal stimuli. The cerebral association cortex and the midbrain have also been reported to process multimodal sensory signals (Stein and Stanford, 2008). Studies of neurons in the superior colliculus which receive visual and auditory afferents (Stein and Meredith, 1993) have demonstrated that these neurons are responsible for integrating information from multiple sensory modalities. These neurons generate highly enhanced responses to spatiotemporally concordant multimodal stimuli derived from the same event, manifesting as several hundred percent increase in the firing rate, relative to the firing rates observed in response to stimulus from any single modality (Wallace et al., 1998).

Neural recordings from multisensory neurons demonstrate that synaptic weights can increase or decrease with relative cue reliability (Morgan et al., 2008; Fetsch et al., 2012). This suggests that synaptic plasticity and learning play an important role in multisensory cue integration, and points toward the important role of rich sensorimotor experiences in developing cue integration capabilities. Neurophysiological evidence suggests that multisensory cue integration is a postnatal developmental process driven by sensorimotor experiences that expose the brain to multimodal stimuli. For example, superior colliculus neurons in newborn cats are incapable of multisensory integration; likely causes of this are the yet-to-develop responsiveness to multisensory inputs (Stein et al., 1973) or the yet-to-develop ability to synthesize multisensory inputs (Wallace and Stein, 1997). However, multisensory cue integration develops relatively quickly after birth. For example, superior colliculus neurons in cats denied audio-visual experience by rearing them in the dark are unable to integrate visual and nonvisual inputs (Wallace et al., 2004). This implies that the brain requires considerable exposure to the statistics of crossmodal events *via* sensorimotor experiences, obtained for example *via* spatial localization tasks, to develop multisensory integration capabilities (Wallace and Stein, 1997). Manipulating sensory experience by presenting controlled audio-visual cues in anesthetized, dark-reared cats has been shown to initiate the development of multisensory integration in superior colliculus neurons (Yu et al., 2010). A review of the neural mechanisms underlying the development of multisensory integration can be found in Stein et al. (2014), Cuppini et al. (2018), and Stein and Rowland (2020). Very recently, neurophysiological evidence has been reported that multisensory experience enables the multisensory transform, i.e., the operation by which unisensory signals are converted into multisensory outputs, to utilize a cooperative computation rather than competitive computation (Wang et al., 2020). In other words, absence of multisensory experience results in the weaker unisensory signal being suppressed; a form of competition between the unisensory signals that ultimately suppresses the multisensory response in superior colliculus neurons. On the other hand, exposure to multisensory experience results in a significant enhancement of multisensory responses, suggesting a cooperation between the unisensory signals.

### 1.3. Earlier work and contribution of current study

As mentioned earlier, I and colleagues have previously reported a computational model for multisensory cue integration that combined auditory and visual directional cues on a moment-by-moment basis (Shaikh et al., 2019). The model was embodied in a simulated robotic agent tasked with localizing a moving audio-visual target *via* orientation movements driven by the result of the cue combination. Auditory directional cues were extracted by a previously reported model (Christensen-Dalsgaard and Manley, 2005) of the lizard peripheral auditory system (Wever, 1978). Visual directional cues were extracted as the location of the target inside the visual receptive field, normalized relative to its center. A single multisensory neuron computed the wheel velocities of the robotic agent as a weighted summation of the auditory and visual directional cues, to initiate orientation movements.

In the previously reported model, the synaptic weights corresponding to both auditory and visual directional cues were updated concurrently *via* independent crossmodal as well as intramodal learning rules. This implied that the learning of the synaptic weights of the auditory directional cue was influenced by the dynamics of the visual directional cue (crossmodal learning), as well as by the dynamics of the auditory directional cue itself (intramodal learning). Similarly, the learning of the synaptic weights of the visual directional cue was influenced by the dynamics of the auditory directional cue (crossmodal learning), as well as by the dynamics of the visual directional cue itself (intramodal learning). The model exhibited multisensory phenomena observed in multisensory neurons in the cat superior colliculus such as sub-additivity, additivity, and super-additivity (Stanford et al., 2005). We demonstrated that concurrent intramodal and crossmodal learning improves both the accuracy and precision of multisensory orientation responses in the target tracking task.

In this study, I hypothesize that experience dependent crossmodal synaptic plasticity may be a plausible mechanism underlying development of multisensory cue integration. I test this hypothesis *via* a computational model of a single multisensory neuron that implements Bayesian multisensory cue integration by using reliability-based cue weighting. The multisensory neuron combines incoming sensory cues *via* a weighted summation scheme, where each cue is weighted by a synaptic weight. The model uses crossmodal synaptic plasticity rules to capture stimulus statistics within the synaptic weights, which are adapted in real time to reflect the relative reliabilities of the participating stimuli. The model is embodied in a simulated robotic agent that learns to localize an audio-visual target by integrating its spatial location, extracted from of auditory and visual sensory modalities. I employ the same visual and auditory cue extraction methodology and the same computational model as in the previous study, but with a slight modification. I simplify the model by removing the intramodal learning rules, such that the model presented here only uses crossmodal learning rules to learn the synaptic weights for each modality. I argue that although intramodal learning improves the accuracy and precision of multisensory orientation responses, it is not required to learn a representation of stimulus statistics of one modality relative to those of another modality. This is because the intramodal learning rules employed in the previous model do not incorporate any information about the other modality. For example, the intramodal learning rule that updates the synaptic weight for the visual modality only uses the dynamics of the visual directional cue (Shaikh et al., 2019).

The remainder of this article is structured in the following manner. I describe the extraction of the visual and auditory directional cues as well as the lizard peripheral auditory system model and its response characteristics in Section 2. I also present the computational model, its operation, and the experimental setup in Section 2. I present and discuss the simulation results in Section 3. I summarize the findings in Section 4.

## 2. Materials and methods

This section describes the extraction of directional cues from the auditory and visual modalities, the computational model of the neural circuit, its embodiment in a simulated agent and the experimental setup.

### 2.1. Extracting directional cues

The elementary neural processes of target detection and recognition in the visual and auditory modalities are not explicitly modeled here, for the sake of simplicity as well as to maintain focus on the multisensory cue integration in the neural processing pipeline.

**Figure 1:**
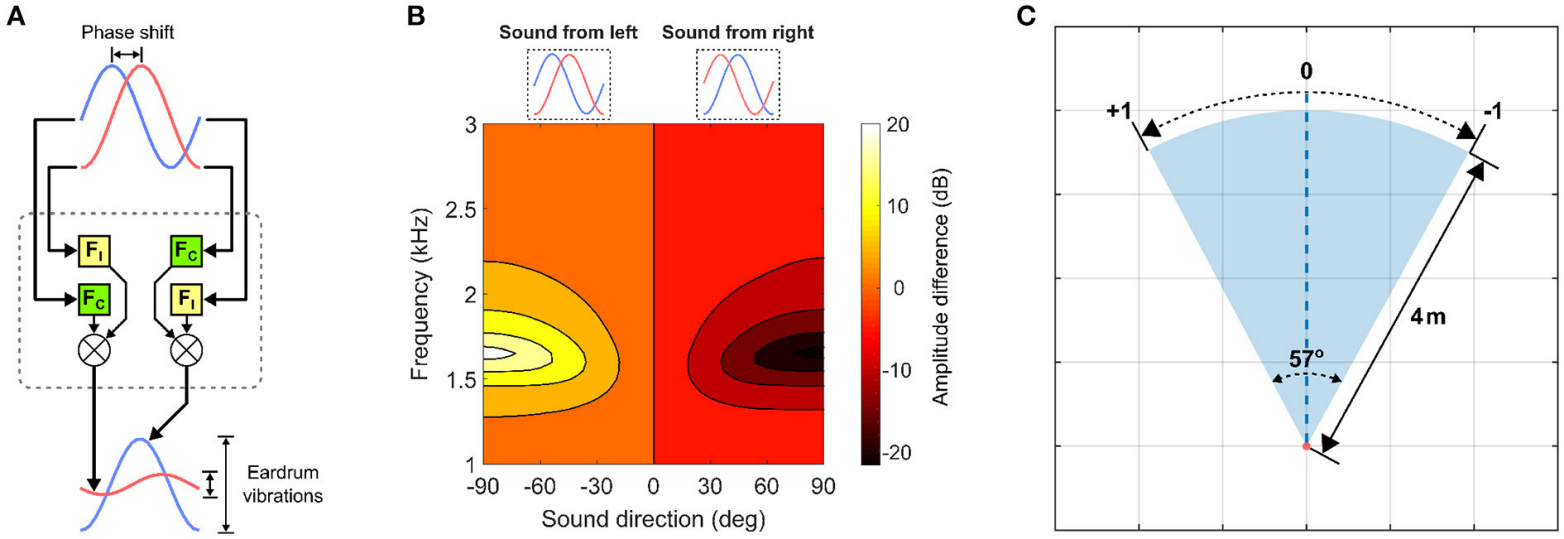
Visual and auditory directional cues. **(A)** The lizard peripheral auditory system model. It filters raw incoming sound signals *via* 4th-order digital bandpass filters F_C_ and F_I_ and generates two outputs representing eardrum vibrations. F_C_ and F_I_ are derived from laser vibrometry measurements of eardrum vibrations (Christensen-Dalsgaard and Manley, 2005). **(B)** Target auditory directional cue determined as a difference in signal power between the model's two outputs. The y-axis corresponds to frequency of sinusoids given as input to the model. **(C)** Encoding of visual directional cue in the visual receptive field.

The auditory directional cue represents the spatial location of a target inside the auditory receptive field. This cue is extracted *via* a computational model of the lizard peripheral auditory system (Figure 1A). This model is used solely due to its availability. The lizard peripheral auditory system is able to detect minute phase differences corresponding to micro-second scale interaural time differences (ITDs) between sound waves arriving externally at either eardrum. These phase differences encode information about relative sound direction and are translated into relatively large differences in the magnitude of eardrum vibrations. These vibrations correspond to perceived sound amplitude at the eardrums. This biophysical conversion is accomplished by highly specialized acoustic filtering performed by the structure of peripheral auditory system (Christensen-Dalsgaard and Manley, 2005). The structural properties of the peripheral auditory system naturally vary across lizard species, and for the specific system being used in this study, have been experimentally determined in previous studies for a tokay gecko specimen. The associated computational model of the peripheral auditory system being used here responds to sound waves of wavelengths 340–85 mm, corresponding to frequencies of 1–4 kHz, with peak responses at approximately 2.2 kHz. Details of the peripheral auditory system, its computational model and response characteristics can be found in Shaikh (2012). The model's output is essentially the difference in perceived sound amplitude at either eardrum and is used as the auditory directional cue (Figure 1B). The auditory receptive field is also assumed to lie within a head-centered reference frame. Thus, the auditory directional cue is zero when the target is in the center of the auditory receptive field and varies non-linearly with the range [−1, +1] relative to the center of the auditory receptive field.

The visual directional cue represents the spatial location of a target inside the visual receptive field (Figure 1C) relative to its center. The visual receptive field is 57° wide and 4 m deep. These values match that of the field-of-view of a standard Microsoft Kinect V1 camera sensor, as this sensor will be used for testing the proposed computational model in subsequent multisensory experiments in a real-world setting for robotic applications. The visual directional cue is determined geometrically when the target is inside the visual receptive field and set to zero when the target is outside the visual receptive field. I assume the visual receptive field to lie within a head-centered reference frame, and thus when the target is inside the visual receptive field the visual directional cue lies within the range [−1, +1] relative to the center of the visual receptive field, wherein the visual directional cue is zero. The right and left boundaries of the visual receptive field respectively correspond to visual directional cues of +1 and −1. The relative position of the target inside the visual receptive field is mapped one-to-one to a corresponding angular position between the two extremes. There is however, a subtle difference between the visual directional cue when the target is at the center of the visual receptive field and that when the target is outside the visual receptive field. In the former case, the visual directional cue is never exactly zero, since white Gaussian noise is added to it to simulate a noisy cue as described in detail in Section 2.3. In the latter case, the visual directional cue is exactly zero, to simulate a target that is not detected.

Normalizing the visual directional cue around the center of the visual receptive field such that the cue is zero when the target is at the center is not strictly necessary. It is done purely for the sake of convenience. However, this choice does have implications on the convergence (i.e., stabilization) of the synaptic weights by the learning rules presented in Section 2.4. These implications are discussed at the end of Section 2.5, where I explain the learning mechanism.

### 2.2. Robotic implementation

**Figure 2:**
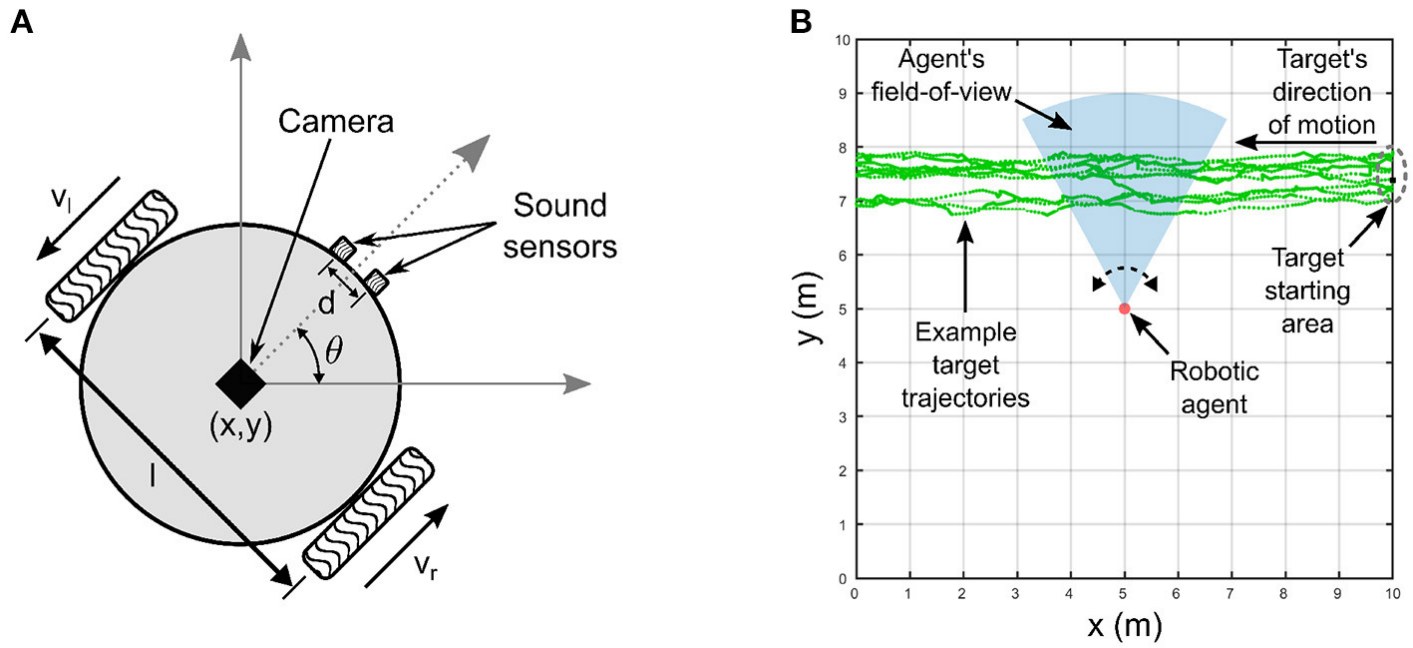
Experimental setup. **(A)** Robotic agent with non-holonomic kinematics. The linear separation *l* between the wheels is 16 cm. **(B)** Experimental arena in which the robotic agent extracts visual and auditory directional cues encoding the relative location of an audio-visual target that moves along randomly generated trajectories.

The robotic agent (Figure 2A) is modeled as a two-wheeled differential drive robot with non-holonomic kinematic constraints. It has two simulated sound sensors, functionally mimicking a pair of microphones, capture the raw auditory signals emitted by the target for further processing by the peripheral auditory model. The parameters of the peripheral auditory model have been derived for a lizard specimen with an ear separation of 13 mm, and the linear separation between the two sensors is chosen as 13 mm, as well. This match ensures that the actual ITD cues available for target localization in the experiments, and the ITD cues to which the peripheral auditory model is tuned are identical. The peripheral auditory model transforms the ITD cues available from the raw auditory signals into the auditory directional cue *x*_a_. A virtual visual sensor, functionally mimicking a Microsoft Kinect V1 camera, extracts the visual directional cue *x*_v_. The directional cues *x*_v_ and *x*_a_ are fed to the proposed computational model for multisensory cue integration as described in the next section. To simulate the robotic agent's movements, I use the standard forward kinematic model given by (2) for differential drive mobile robots (Dudek and Jenkin, 2010). This model takes as input the robot's current position as coordinates *x* and *y* and current orientation θ in a two-dimensional plane as well as its wheel velocities and generates the new position and orientation of the robot for a given time step δ*t*. These terms together describe the pose [*x, y*, θ] of the robotic agent.


(1) $$\begin{array}{l} \left[\begin{array}{l} \text{x }\\ \text{y}\\ \theta \end{array}\right]=\left[\begin{array}{l} cos(\omega \delta t)-sin(\omega \delta t)0\\ sin(\omega \delta t)-cos(\omega \delta t)0\\ \text{ 0 0 1} \end{array}\right]\left[\begin{array}{l} Dsin(\theta )\\ -Dcos(\theta )\\ \;0 \end{array}\right]\\ \;+[\begin{array}{l} x-Dsin(\theta )\\ y+Dcos(\theta )\\ \;\omega \delta \end{array}] \end{array}$$

$$\begin{array}{r} \text{where, angular velocity}\omega =\frac{\left(v_{\text{r}}-v_{\text{l}}\right)}{l}\text{, and}\\ \text{distance}D\text{from instantaneous}\\ \text{center of curvature}=\frac{l}{2}\frac{\left(v_{\text{r}}+v_{\text{l}}\right)}{\left(v_{\text{r}}-v_{\text{l}}\right)} \end{array}$$

### 2.3. Experimental setup

The multisensory cue integration process executed by the proposed computational model is realized in the context of an audio-visual tracking task in simulation. The robotic agent tracks an audio-visual target that travels along unknown, randomly generated motion paths by orienting itself toward the target *via* on-the-spot rotational movements (Figure 2B). The goal of the robotic agent in the tracking task is to attempt to maintain the target within the centers of both the auditory and visual receptive fields. The target moves from the right side of the robotic agent to the left. The target's linear velocity along the direction of motion varies randomly between 0 and 10 m/time step a random number of times, and that perpendicular to the direction of motion varies randomly between 0 and 4 m/time step. Each time the velocity along the direction of motion is modified, the number of simulation time steps until the next change in velocities occurs is also randomly varied between 5 and 10 time steps. Similarly, each time the velocity perpendicular to the direction of motion is modified, the number of simulation time steps until the next change in velocities occurs is also randomly varied between 0 and 5 time steps. This strategy generates random zig-zag motion paths with randomly and independently varying velocities both along and perpendicular to the direction of motion. This allows the robotic agent to simulate intermittent movements prevalent in real world scenarios, for example a predator tracking a moving prey. The target emits two 2.2 kHz sinusoidal tones with identical and fixed peak-to-peak amplitudes as a binaural auditory signal. This signal serves as input to the peripheral auditory model, functionally mimicking the two separate paths of different path lengths traveled by the sound waves from the target to either sound sensor on the robotic agent. The difference in path lengths traveled by the two sinusoids results in a phase difference δϕ in radians calculated as


(2) $$\begin{array}{r} \delta \phi =2\pi \cdot f\cdot \frac{\text{d}\cdot sin\left(\theta \right)}{\text{c}}, \end{array}$$

where the frequency of the input sinusoids is *f* = 2.2 kHz, the physical separation between the two sound sensors is d = 13 mm and the speed of sound in air is c = 340 m/s. Sound arrives at the two sound sensors from the heading θ relative to the frontal axis of the robotic agent. The difference in path lengths of the sound signals arriving at the sound sensors is given by d·*sin*(θ).

A larger physical separation between the sound sensors will generate correspondingly greater ITD cues. However, increasing the physical separation does not lead to a larger phase difference in (2). The maximum possible phase difference is attained when the sound arrives from the left or right extremes relative to the sound (θ = ±90°). The peak binaural difference response in the peripheral auditory system, given a physical sensor separation of d = 13 mm, is obtained for a sound frequency *f* = 1.5 kHz (Christensen-Dalsgaard and Manley, 2005). Substituting these values for sound frequency, sound direction and physical separation in (2), the maximum possible phase difference can be determined as $\delta \phi =2\pi \cdot 1.5\text{kHz}\cdot \frac{13\text{mm}\cdot sin\left(\pm 90^{∙}\right)}{340,000\text{mm}/\text{s}}\equiv \pm 0.36036$ radians. The sound frequency that generates peak binaural difference response in the peripheral auditory model, given a physical separation of d = 100 mm, can be determined as $f=1.5\text{kHz}\cdot \frac{13\text{mm}}{100\text{mm}}=0.195\text{kHz}$. Substituting these values for sound frequency, sound direction and physical separation in (2), the maximum possible phase difference can be determined as $\delta \phi =2\pi \cdot 0.195\text{kHz}\cdot \frac{100\text{mm}\cdot sin\left(90^{∙}\right)}{340,000\text{mm}/\text{s}}\equiv 0.36036$ radians. Therefore, using a physical separation >13 mm offers no distinct advantage in localization.

Sound level differences between the two sound sensors are also significant for localization, but only if a physical obstruction with dimensions greater than the half-wavelength of the sound frequency in question, or if the sound sensors are significantly far apart from each other. I assume neither of the two possibilities and thus sound level differences between the sound signals arriving at the sound sensors are assumed to be non-existent. Interaural time and level difference cues are significant for lateral sound localization in the azimuth plane but are insufficient for resolving sound sources located directly in the front or the back, as the ITD and ILD cues are identical in these situations. This front-back ambiguity can be resolved by spectral filtering of incoming sound by the pinnae (Batteau, 1967). However, in my experimental setup, I focus only on lateral sound localization in the frontal semi-circle in the azimuth plane, and therefore spectral filtering effects of pinnae ate not modeled. I argue that modeling such intricate pre-processing is also not necessary to validate the hypothesis.

The target emits intermittent sound signals, with a random duty cycle i.e., the sound emission is off for a random number of simulation time steps between 5 and 10 and on for a random number of simulation time steps between 10 and 15. Visual detection only occurs when the target is inside the field-of-view, and no visual detection is triggered when it is outside the field-of-view. White Gaussian noise is added to both the visual and auditory directional cues, and the signal-to-noise ratios for both cues can be manipulated independently to simulate relatively low or high cue reliability. This strategy simulates noisy auditory and visual detection events typically observed in real-world situations.

**Figure 3:**
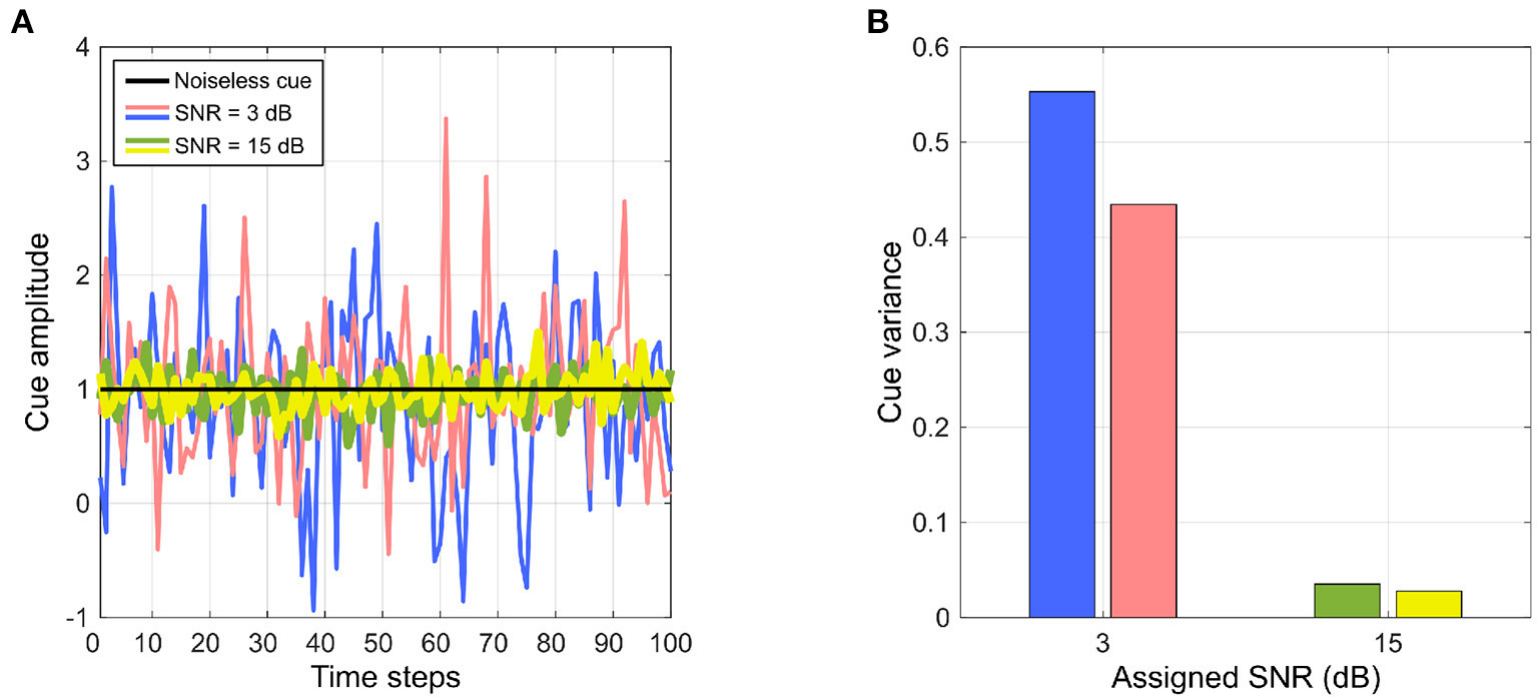
Relationship between assigned SNR used to generate cues and cue variance. **(A)** An “ideal” cue with zero noise (in black) and generated instances of the same cue with added white Gaussian noise with assigned SNRs. Noisy cue instances in red and blue both are generated with a SNR of 3 dB. Noisy cue instances in green and yellow both are generated with a SNR of 15 dB. **(B)** Variance of the noisy cues. Two noisy instances of the same cue generated with the same assigned SNR can exhibit different variances.

The experimental simulations are implemented in MATLAB R2021b (Mathworks Inc.). The SNRs in the auditory and visual directional cues were manipulated *via* MATLAB's built-in function *awgn* (*x, snr, signalpower*) implemented in the MATLAB Communications toolbox. This function adds white Gaussian noise to a given signal, and takes three arguments—the signal *x* to which noise must be added, the desired SNR *snr* in *x* and a third parameter *signalpower* set to “*measured”*, which computes the signal level of *x* to determine the appropriate noise level to be added, based on the specified value of *snr*. The argument *snr* is assigned a value to manipulate the noise level in the signal and achieve a targeted SNR. Increasing the SNR reduces the noise levels in the signals, and thus reduces the variance of the signal as well. Since normalized cue reliability is inversely proportional to the cue variance as evident in (5), increasing the SNR should consequently decreases the variance and increases the normalized reliability. However it must be noted that the noise introduced by *awgn*() in each trial comes from an independent, randomly generated normal distribution that is used by *awgn*(). The randomly generated normal distributions use MATLAB's random number generator that is initialized with a random seed for each trial. This implies that the noise added to the auditory and visual cues in each trial comes from a different, random underlying distribution, and therefore changes in the SNR values of the auditory and visual cues may not directly correspond to equivalent changes in cue variances (and thus reliabilities). In other words, it is possible that different instances where a cue assigned the same SNR exhibits a different variance, which allows for trial-to-trial variability in the cues. An example of such differences in cue variance can be seen in Figure 3, where four noisy instances of an “ideal” cue are generated using *awgn*(). Two of the instances are generated using the same SNR of 3 dB, and the other two instances are generated using the same SNR of 15 dB.

The SNR of the auditory cue is manipulated by adding white Gaussian noise, as described in the previous paragraph, directly to the raw sinusoidal signals emitted by the target. The computational model of the lizard peripheral auditory system does not incorporate any noise filtering. Noise in the raw sinusoidal inputs to the model is therefore passed along unaltered to the auditory directional cue as noisy estimates of relative target location as the model's output. The SNR of the visual cue is manipulated by adding white Gaussian noise directly to the visual directional cue. This is in contrast to conventional approaches in manipulating SNR in visual stimuli by adding visual artifacts of known intensity or coherence as background noise. Due to the absence of a visual processing pipeline, such background noise is assumed to be directly encoded in the visual directional cue as noisy estimates of relative target location. Vision is generally more adaptable and robust to external noise such as contrast changes, thanks to automatic gain control and selective attention mechanisms in the brain, than audition. Therefore, visual motion processing and target location estimation is also generally more adaptable than auditory motion processing and location estimation. Based on this, I argue that the computational burden of simulating the physics of raw visual signal generation and transmission as well as simulating a visual processing pipeline solely to manipulate SNR of the visual signal by adding noise to the raw visual signal is not strictly required to test the proposed hypothesis. Instead, I assume that the noise in the raw visual signal is reflected directly in the visual directional cue as noisy estimates of relative target location.

### 2.4. Computational model

**Figure 4:**
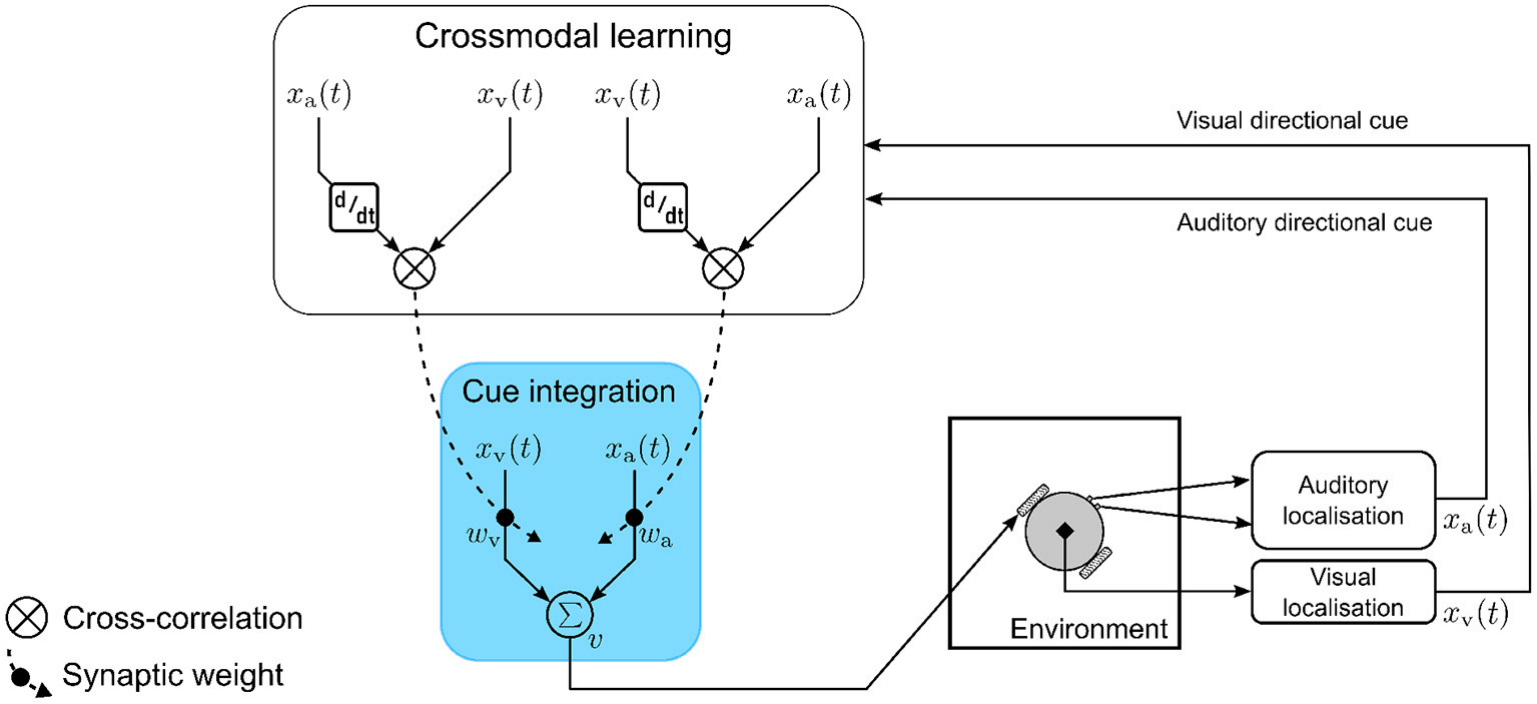
The computational model for multisensory cue integration. The model is embodied in a robotic agent that extracts auditory and visual directional cues pertaining to the location of an audio-visual target. The cues are weighted by synaptic weights and are integrated together by a single multisensory neuron to compute motor orientation responses (blue box). The synaptic weights are adapted on a moment-by-moment basis *via* crossmodal learning (white box) that is driven by temporal correlation between the auditory and visual directional cues.

The computational model (Figure 4) consists of a single multisensory neuron receiving inputs as auditory and visual directional cues *x*_a_ and *x*_v_, respectively. The auditory and visual directional cues are respectively weighted by synaptic weights *w*_a_ and *w*_v_ before being integrated within the multisensory neuron. At each simulation time step *t*, the multisensory neuron computes a motor velocity |*v*| as the weighted sum of auditory and visual directional cues, respectively. Multisensory cue integration in the neuron is therefore modeled as


(3) $$\begin{array}{r} |v|=w_{\text{v}}\cdot x_{\text{v}}\left(t\right)+w_{\text{a}}\cdot x_{\text{a}}\left(t\right). \end{array}$$

The computed motor velocity |*v*| is assigned to the individual motor velocities |*v*_l_| and |*v*_r_| for the left and right wheels, respectively of the robotic agent such that |*v*_l_| = |*v*_r_| = |*v*|. The signs for *v*_l_ and *v*_r_ indicating direction of wheel rotation are then assigned according to the direction of rotation of the robotic agent. This reactive strategy essentially generates a reflexive orientation response to stimulus onset, irrespective of stimulus modality. The robotic agent therefore reacts immediately to a sensory detection event in either modality by attempting to orient itself toward the relative direction of the target. A visual detection event occurs when the target is in the visual receptive field, and an auditory detection event occurs when the target emits a sound.

I define two crossmodal learning rules, one updating the synaptic weight *w*_a_ and the other updating the synaptic weight *w*_v_. Both rules are based on the input correlation (ICO) learning rule (Porr and Wörgötter, 2006) where synaptic weights are updated proportional to the temporal correlation between the inputs. Correlation has been suggested as a general mechanism for multisensory integration (Parise and Ernst, 2016). The ICO learning rule is an unsupervised, closed-loop learning algorithm adapted from differential Hebbian learning (Kosko, 1986; Klopf, 1988). There is evidence to suggest that multisensory integration is an adaptive mechanism (Stein and Rowland, 2011). The correlation based learning utilized here is a form of associative learning and is therefore a feasible mechanism underlying synaptic plasticity. The learning rule models heterosynaptic plasticity, in that the synaptic strength between a pre-synaptic neuron and a post-synaptic neuron is modified in response to temporally correlated activity (with respect to the pre-synaptic neuron) of a third pre-synaptic neuron.

Modeling the crossmodal learning as heterosynaptic plasticity serves two functions—(a) it ensures that information encoded in the synaptic weights about the sensory stimuli are shared across the two modality-specific processing pathways, and (b) it acts a regulatory mechanism to ensure that the synaptic weights converge without catastrophic forgetting. In the model, the post-synaptic neuron is the multisensory neuron, while I assume that the visual and auditory directional cues are the respective outputs of two pre-synaptic neurons that are not explicitly modeled for the sake of simplicity. The two crossmodal learning rules are mathematically formulated as


(4) $$\begin{array}{r} \delta w_{\text{a}}=\mu \cdot x_{\text{a}}\left(t\right)\cdot \frac{\delta x_{\text{v}}\left(t\right)}{\delta t}\text{and}\delta w_{\text{v}}=\mu \cdot x_{\text{v}}\left(t\right)\cdot \frac{\delta x_{\text{a}}\left(t\right)}{\delta t}. \end{array}$$

Both learning rules employ the same fixed learning rate μ. The learning rate proportionally influences the amount of synaptic weight update, i.e., higher learning rates imply larger weight updates and vice versa. Larger synaptic weights in general imply higher values for motor velocity |*v*|, resulting in higher rotational speeds and thus faster orientation responses. A large |*v*| also implies a large amount of rotation and if |*v*| becomes too large it will result in large orientation responses which may cause the robotic agent's heading to overshoot beyond the target's direction. The agent will then rotate in the opposite direction by a large amount to correct for the overshoot. This can introduce undesirable oscillations in its movements, leading to localization becoming unstable. On the other hand, lower learning rates μ imply small weight updates. This may result in the robotic agent rotating by insignificant amounts and lagging behind the target, leading to undesirable sluggish responses. The learning rate μ is therefore optimized *via* trial and error to minimize oscillations in the orientation response as well as to maximize the speed of orientation response.

The computational model is embodied as the robotic agent placed in a task environment of the experimental setup. Embodying the model in an agent is necessary for performing the tracking task that generates the required sensorimotor experience that drives the crossmodal learning and adapts the synaptic weights. Given that the crossmodal learning rules while being independent in operation essentially share the same synaptic weights, they influence the results of each other's operation in a complementary manner.

### 2.5. Model operation and learning

Irrespective of whether the target is inside the visual receptive field or not, the auditory cue weight *w*_a_ is updated only when the target emits a sound signal. The visual cue weight *w*_v_ is updated only when the target is inside the visual receptive field.

The auditory cue weight update is driven by computing the cross-correlation between the auditory directional cue and the first-order time derivative of the visual directional cue. The result of this cross-correlation determines the amount by which the weight is updated. The correlation is greater when the target is at the extremes of the auditory receptive field and the visually perceived target motion is fast, resulting in larger weight updates. The correlation is smaller when the target is closer to the center of the auditory receptive field and the visually perceived target motion is slow, resulting in smaller weight updates. Thus, the weight updates are dependent on the distance of the target from the center of the auditory receptive field and the visually perceived speed of the target. This mechanism ensures that the weight updates progressively get relatively smaller the closer the target moves to the center of the auditory receptive field and the slower it moves. This allows the weights to stabilize when the target is in the center of the auditory receptive field, i.e., when the robot is pointing directly toward the target, and consequently the learning to converge.

The visual cue weight update is driven by computing the cross-correlation between the visual directional cue and the first-order time derivative of the auditory directional cue. The result of this cross-correlation determines the amount by which the weight is updated. The correlation is greater when the target is at the extremes of the visual receptive field and the acoustically perceived target motion is fast, resulting in larger weight updates. The correlation is smaller when the target is closer to the center of the visual receptive field and the acoustically perceived target motion is slow, resulting in smaller weight updates. Thus, the weight updates are dependent on the distance of the target from the center of the visual receptive field and the acoustically perceived speed of the target. This mechanism ensures that the weight updates progressively get relatively smaller the closer the target moves to the center of the visual receptive field and the slower it moves. This allows the weights to stabilize when the target is in the center of the visual receptive field, i.e., when the robot is pointing directly toward the target, and consequently the learning to converge.

It is evident from (4) that the synaptic weight corresponding to any modality only stabilize when the signal in that modality is either zero or is constant with respect to time. This is because under either of these conditions, the derivative terms in (4) become zero. Recall that the behavioral goal of the robotic agent in the tracking task is to attempt to maintain the target within the centers of both the auditory and visual receptive fields. This implies that the synaptic weights should stabilize when this condition is reached. Therefore, one could also set the sensory signals to have non-zero values at the centers of the receptive fields, as it would still satisfy the condition for weight convergence.

### 2.6. Experimental design

I perform two sets of trials to test the hypothesis that experience dependent crossmodal synaptic plasticity may be a plausible mechanism underlying the development of multisensory cue integration in the form of a maximum-likelihood estimate. In the first set of trials, I test the hypothesis by allowing the embodied computational model to learn modality-specific synaptic weights while performing the audio-visual tracking task. The signal-to-noise ratio (SNR) of the visual directional cue SNR_v_ is kept fixed at 3 dB, and the SNR of the auditory directional cue SNR_a_ is varied within the range [6–21] dB, in steps of 3 dB. This allows one to explore how the synaptic weights adapt to the relative noise level in the stimulus. The robotic agent performs 20 such trials one after the another. I observe whether the development of the weights reflects the relative reliabilities (as represented by the inverse of the variance) of the auditory and visual cues, thereby capturing the stimulus statistics. This set of 20 trials is then repeated in identical manner, but in this instance the SNR of the auditory directional cue SNR_a_ is kept fixed at 3 dB, and the SNR of the visual directional cue SNR_v_ is varied in the range [6–21] dB, in steps of 3 dB. This allows one to test for any modality-specific bias in the multisensory cue integration. For all trials, the learning rate μ is set to 0.09, and the initial values of the synaptic weights are randomly set to *w*_v_ = *w*_a_ = 0.1. The synaptic weights are not reset between trials, i.e., the agent starts each trial with the synaptic weights learned in the previous trial. The robotic agent initially points straight ahead in all trials. In both sets of trials, the relative sensory cue reliabilities (inverse of the variance) for the auditory and visual cues are determined as normalized reliabilities given respectively by


(5) $$\begin{array}{r} \frac{1}{\sigma_{a_{norm}}^{2}}=\frac{\frac{1}{\sigma_{a}^{2}}}{\frac{1}{\sigma_{a}^{2}}+\frac{1}{\sigma_{v}^{2}}}\text{and}\frac{1}{\sigma_{v_{norm}}^{2}}=\frac{\frac{1}{\sigma_{v}^{2}}}{\frac{1}{\sigma_{a}^{2}}+\frac{1}{\sigma_{v}^{2}}}, \end{array}$$

where $\sigma_{a}^{2}$ and $\sigma_{v}^{2}$ are the respective variances of the auditory and visual cues. The cue variances are determined *via* the built-in MATLAB function *var*().

In the second set of trials, the SNRs for both modalities are kept identical and varied simultaneously in the range [3–21] dB, in steps of 3 dB. This allows one to explore how the development of modality-specific synaptic weights is affected by overall noise levels in the stimuli, and to test for any noise-dependent bias in the multisensory cue integration. Twenty such trials are performed, one after the other. For all trials, the learning rate μ is set to 0.09 and the initial values of the synaptic weights are randomly set to *w*_v_ = *w*_a_ = 0.1. The synaptic weights are not reset between trials, i.e., the agent starts each trial with the synaptic weights learned in the previous trial. The robotic agent initially points straight ahead in all trials. An example set of 20 trials along with the evolution of synaptic weights can be seen in the video file “video.mp4” in the Supplementary materials.

## 3. Results and discussion

**Figure 5:**
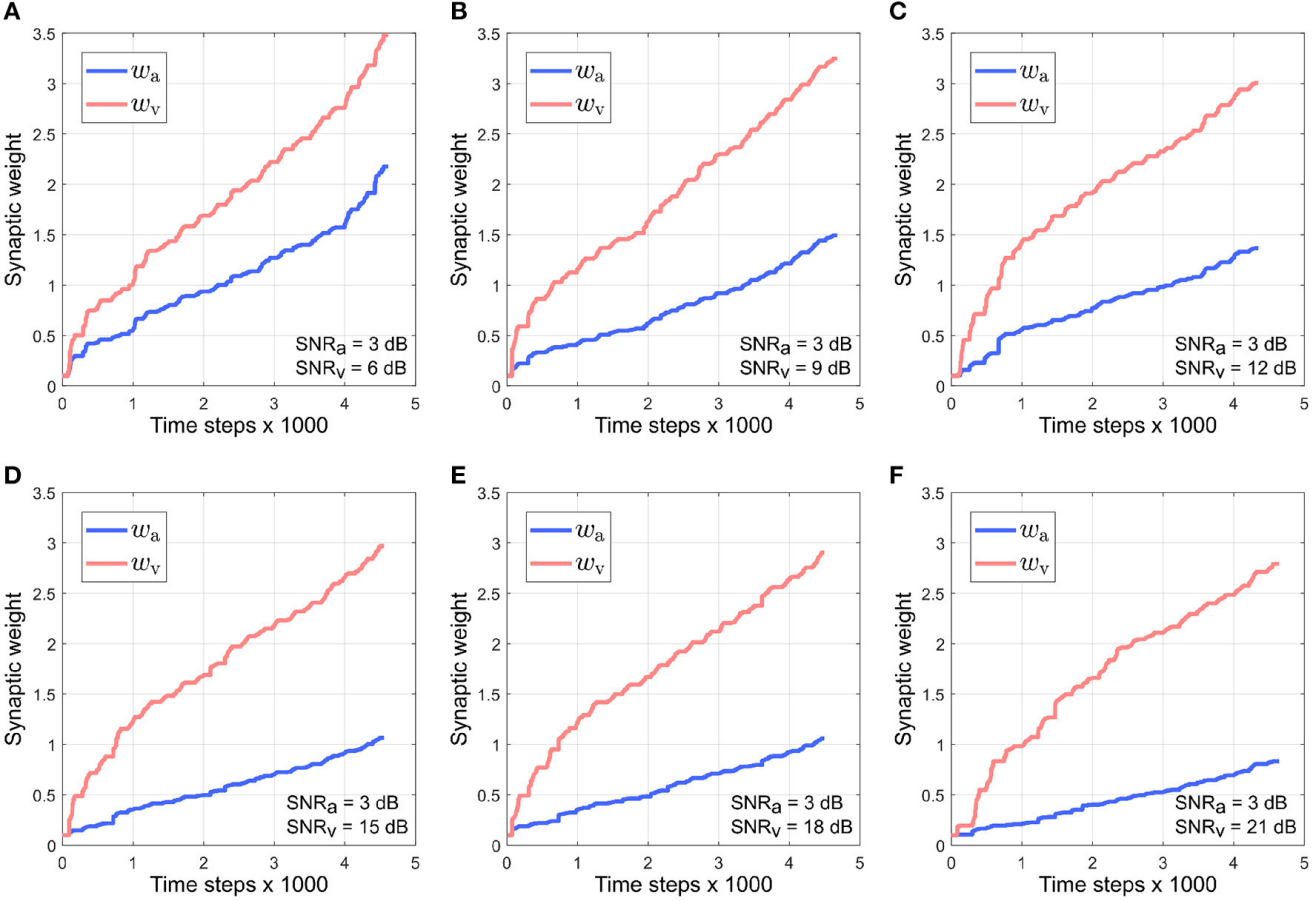
Evolution of synaptic weights *w*_a_ and *w*_v_ through the course of the trials. The SNR for the auditory directional cue SNR_a_ is kept fixed at 3 dB and the SNR for the visual directional cue SNR_v_ is varied from 6 to 21 dB **(A–F)** in steps of 3 dB.

Figure 5 depicts the evolution of the synaptic weights when the SNR for the auditory directional cue *x*_*a*_ is kept fixed at 3 dB and the SNR for the visual directional cue *x*_*v*_ is varied between 6 and 21 dB (Figures 5A–F). The synaptic weight *w*_*v*_ of the visual directional cue quickly rises above the synaptic weight *w*_*a*_ of the auditory directional cue in all cases during the course of the trials. Since the auditory cue is nosier than the visual cue in all the cases, it exhibits relatively larger variations in amplitude than the visual cue. This implies that the time derivative $\frac{dx_{a}}{dt}$ is relatively larger than the time derivative $\frac{dx_{v}}{dt}$, as well as relatively larger than the instantaneous values of both *x*_*a*_ and *x*_*v*_. In other words, the dynamics of the auditory directional cue *x*_*a*_, encoded in its time derivative, are stronger than the dynamics of the visual directional cue *x*_*v*_ (encoded in its time derivative) as well as larger than the instantaneous values of both the cues *x*_*a*_ and *x*_*v*_. As given by the equations in (4), the synaptic weight update $\frac{dw_{v}}{dt}$ for the visual directional cue *x*_*v*_ is dependent on *x*_*v*_ and $\frac{dx_{a}}{dt}$ and the synaptic weight update $\frac{dw_{a}}{dt}$ for the auditory directional cue *x*_*a*_ is dependent on *x*_*a*_ and $\frac{dx_{v}}{dt}$. The relatively stronger dynamics of *x*_*a*_ results in the synaptic weight updates $\frac{dw_{v}}{dt}$ for the visual directional cue being relatively larger than the synaptic weight updates $\frac{dw_{a}}{dt}$ for the auditory directional cue. Consequently, the synaptic weight *w*_*v*_ of the visual directional cue quickly rises above the synaptic weight *w*_*a*_ of the auditory directional cue.

**Figure 6:**
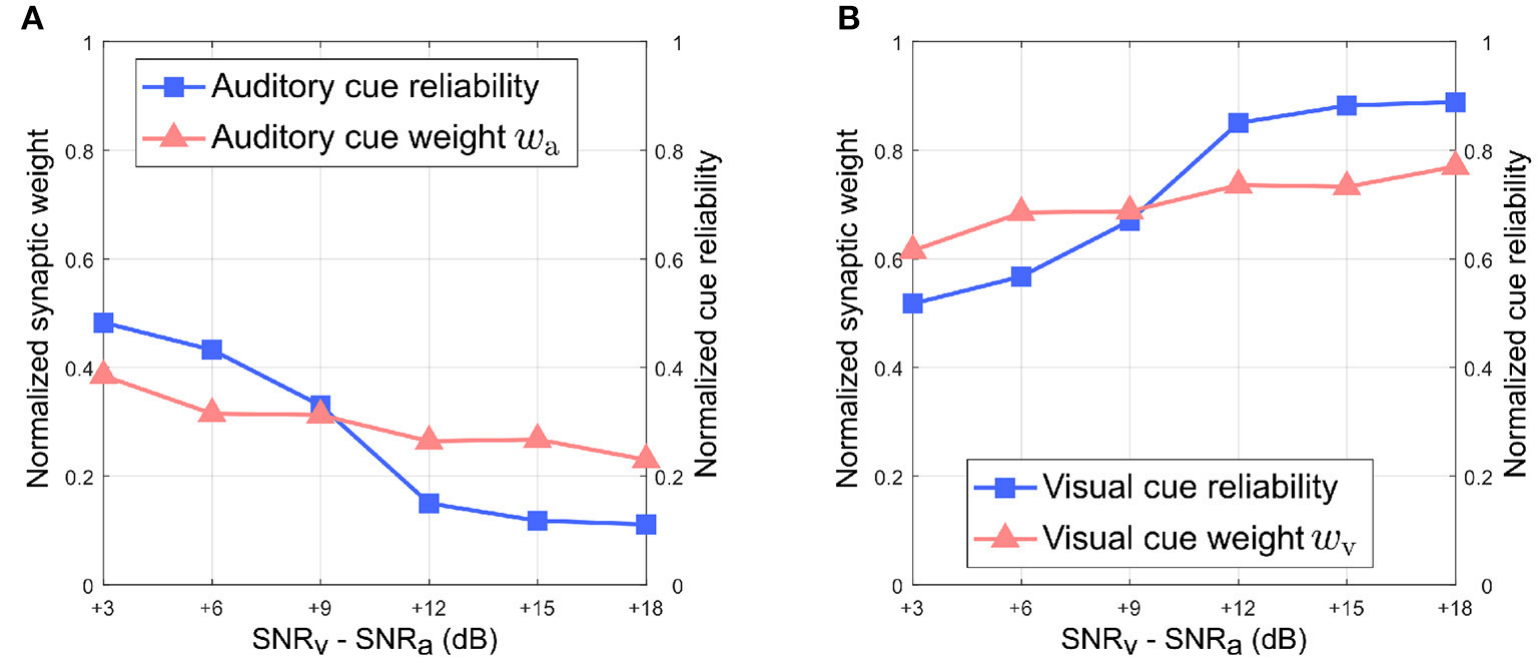
Normalized synaptic weights and normalized cue reliabilities vs. relative cue noise levels when auditory directional cue noise is greater than visual directional cue noise. **(A)** Audition. **(B)** Vision. The SNR for the auditory directional cue SNR_a_ is kept fixed at 3 dB and the SNR for the visual directional cue SNR_v_ is varied from 6 to 21 dB in steps of 3 dB.

Figure 6 depicts the relationship between the normalized synaptic weights as well as the normalized cue reliabilities, and the difference in noise levels between the auditory and visual directional cues when the auditory directional cue is noisier than the visual directional cue. The normalized synaptic weight *w*_*a*_ for the auditory directional cue, learned after 20 trials, lies relatively close to the normalized auditory cue reliability in all cases (Figure 6A). However, there is a finite offset between normalized *w*_*a*_ and normalized cue reliability, and additional trials do not reduce or eliminate this offset. Similarly, the normalized synaptic weight *w*_*v*_ for the visual directional cue, learned after 20 trials, lies relatively close to the normalized visual cue reliability in all cases (Figure 6B). Once again, there is a finite offset between normalized *w*_*v*_ and normalized cue reliability, and additional trials do not reduce or eliminate this offset.

Overall, normalized *w*_*a*_ is relatively smaller than normalized *w*_*v*_ in all cases. This observation supports the reliability-based cue weighting scheme because the relative reliability of the auditory cue is set to be smaller than that of the visual cue, which implies that normalized *w*_*a*_ should be lower than normalized *w*_*v*_. This is because the learned synaptic weight *w*_*a*_ is smaller than the learned synaptic weight *w*_*v*_ in all cases. As the relative noise level of the visual directional cue decreases, i.e., as its relative reliability increases, the normalized synaptic weight *w*_*v*_ of the visual directional cue increases as well. Conversely, the normalized synaptic weight *w*_*a*_ of the auditory directional cue decreases when the relative noise level of the visual cue decreases, i.e., its relative reliability increases. This indicates that the relative synaptic weights follow relative cue reliability, allowing for dynamic re-weighting of synaptic weights.

**Figure 7:**
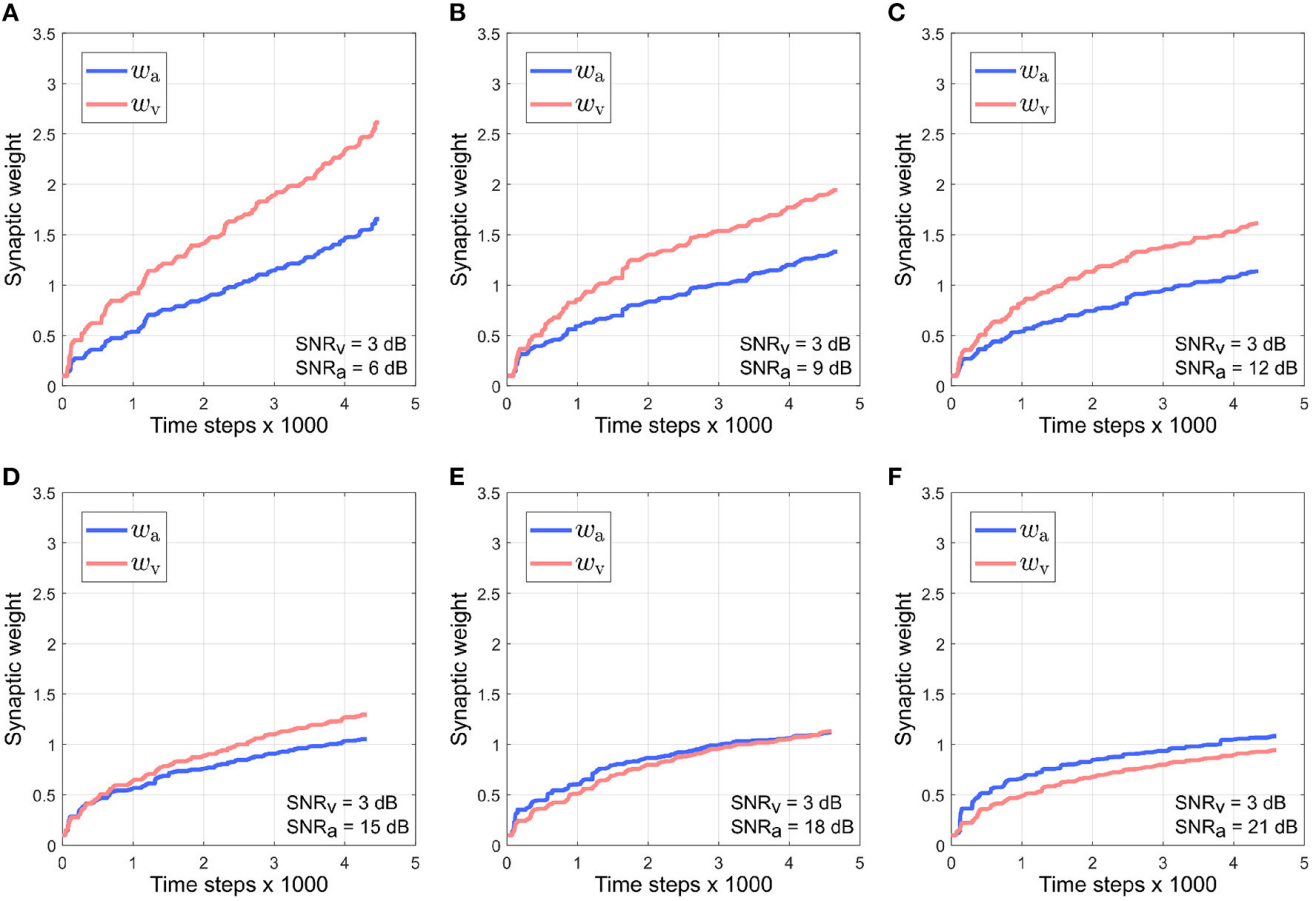
Evolution of synaptic weights *w*_a_ and *w*_v_ through the course of the trials. The SNR for the visual directional cue SNR_v_ is kept fixed at 3 dB and the SNR for the auditory directional cue SNR_a_ is varied from 6 to 21 dB **(A–F)** in steps of 3 dB.

Figure 7 depicts the evolution of the synaptic weights when the SNR for the visual directional cue *x*_*v*_ is kept fixed at 3 dB and the SNR for the auditory directional cue *x*_*a*_ is varied between 6 and 21 dB (Figures 7A–F). The synaptic weight *w*_*v*_ of the visual directional cue still quickly rises above the synaptic weight *w*_*a*_ of the auditory directional cue during the course of the trials when the difference in noise levels between the auditory cue and visual cue is below +15 dB (Figures 7A–D). When the difference in noise levels between the auditory cue and visual cue is +15 dB and above, the synaptic weight *w*_*a*_ of the auditory directional cue begins to rise above the synaptic weight *w*_*v*_ of the visual directional cue (Figures 7E,F). Since the visual cue is nosier than the auditory cue in all cases, it exhibits relatively larger variations in amplitude than the auditory cue. This implies that the time derivative $\frac{dx_{v}}{dt}$ is relatively larger than the time derivative $\frac{dx_{a}}{dt}$, as well as relatively larger than the instantaneous values of both *x*_*a*_ and *x*_*v*_. In other words, the dynamics of *x*_*v*_, encoded in its time derivative, are stronger than the dynamics of *x*_*a*_ (encoded in its time derivative) as well as larger than the instantaneous values of both *x*_*a*_ and *x*_*v*_. As given by the equations in (4), the weight update for *x*_*a*_, $\frac{dw_{a}}{dt}$ is dependent on *x*_*a*_ and $\frac{dx_{v}}{dt}$ and the weight update for *x*_*v*_, $\frac{dw_{v}}{dt}$ is dependent on *x*_*v*_ and $\frac{dx_{a}}{dt}$. The relatively stronger dynamics of *x*_*v*_ results in auditory cue weight updates $\frac{dw_{a}}{dt}$ being relatively larger than the visual cue weight updates $\frac{dw_{v}}{dt}$.

This should result in the synaptic weight *w*_*a*_ of the visual directional cue quickly rising above the synaptic weight *w*_*v*_ of the auditory directional cue, but the opposite effect is observed. Since the auditory cue is intermittent, it does not contribute to auditory cue weight updates when it is zero. The synaptic weight updates for *x*_*a*_, as given by $\frac{dw_{a}}{dt}$ in (4), are dependent on *x*_*a*_ and $\frac{dx_{v}}{dt}$. This implies that $\frac{dw_{a}}{dt}$ is zero when the auditory cue *x*_*a*_ is zero. However, zero instantaneous values do not necessarily imply zero dynamics. For example, when the auditory cue transitions from zero to a non-zero value (or from a non-zero value to a zero value), the time derivative of this transition is non-zero. The synaptic weight updates for *x*_*v*_, as given by $\frac{dw_{v}}{dt}$ in (4), are dependent on *x*_*v*_ and $\frac{dx_{a}}{dt}$. When the auditory cue dynamics are non-zero, then $\frac{dx_{a}}{dt}$ is also non-zero. This implies that $\frac{dw_{v}}{dt}$ can be non-zero even when the auditory cue is zero. The intermittent nature of the auditory cue guarantees a significant number of non-zero transitions and thus significant non-zero dynamics, and this results in the synaptic weight *x*_*v*_ for the visual cue being updated more than the synaptic weight *x*_*a*_ for the auditory cue during the course of each trial.

As the noise levels in the auditory directional cue fall below those in the visual directional cue, the former exhibits progressively weaker variations in its amplitude as compared to the latter. This results in the time derivative $\frac{dx_{a}}{dt}$ of the auditory directional cue becoming progressively smaller than the time derivative $\frac{dx_{v}}{dt}$ of the visual directional cue. This implies that the dynamics of the auditory directional cue become weaker than those of the visual directional cue. When the difference in noise levels between the auditory cue and visual cue is +15 dB and above, the dynamics of the auditory directional cue are likely significantly weaker than those of the visual directional cue. This implies that the synaptic weight updates for *x*_*v*_, which are dependent on *x*_*v*_ and $\frac{dx_{a}}{dt}$, are of lower magnitude than the synaptic weight updates for *x*_*a*_, which are dependent on *x*_*a*_ and $\frac{dx_{v}}{dt}$.

It must be noted that irrespective of which modality is noisier, the instantaneous values of both *x*_*a*_ and *x*_*v*_ only contribute significantly to the synaptic weight updates of their respective modalities when the target is at the peripheries of the respective receptive fields. This is because both *x*_*a*_ and *x*_*v*_ reach maxima when the target is at the peripheries of the respective receptive fields. The integrated output of the computational model is the motor velocity for the robotic agent which drives the orientation movements that bring the target closer to the centers of both the receptive fields. The orientation movements are rapid, which implies that the target spends relatively little time at the peripheries of the visual and auditory receptive fields. This results in a rapid decrease in the instantaneous values of both *x*_*a*_ and *x*_*v*_, resulting in the overall contributions of the instantaneous values of *x*_*a*_ and *x*_*v*_ being significantly weaker as compared to the contributions of their dynamics.

**Figure 8:**
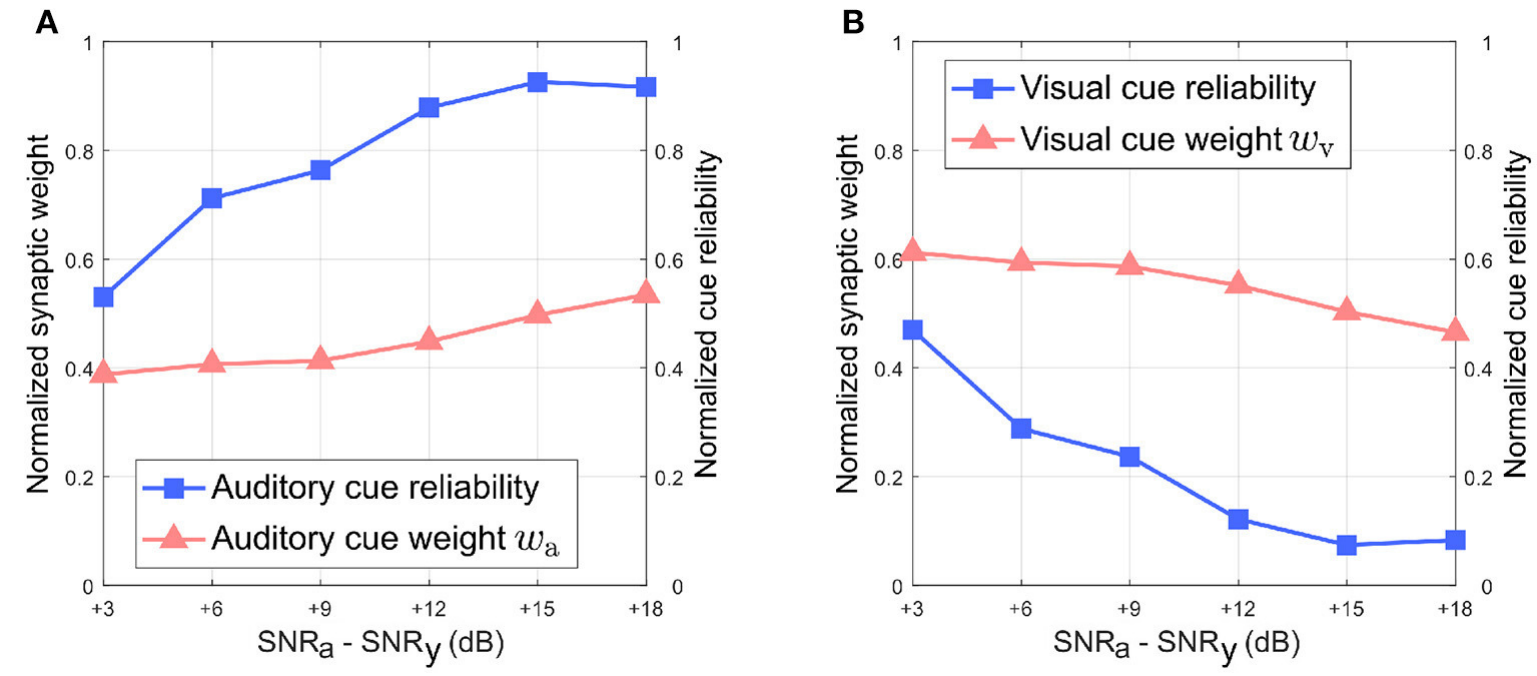
Normalized synaptic weights and normalized cue reliabilities vs. relative cue noise levels when visual directional cue noise is greater than auditory directional cue noise. **(A)** Audition. **(B)** Vision. The SNR for the visual directional cue SNR_v_ is kept fixed at 3 dB and the SNR for the auditory directional cue SNR_a_ is varied from 6 to 21 dB in steps of 3 dB.

Figure 8 depicts the relationship between the normalized synaptic weights as well as the normalized cue reliabilities, and the difference in noise levels between the auditory and visual directional cues when the visual directional cue is noisier than the auditory directional cue. The normalized synaptic weight *w*_*a*_ for the auditory directional cue, learned after 20 trials, lies relatively close to the normalized auditory cue reliability in all cases (Figure 8A). However, there is a finite offset between normalized *w*_*a*_ and the normalized cue reliability, and additional trials do not reduce or eliminate this offset. Similarly, the normalized synaptic weight *w*_*v*_ for the visual directional cue, learned after 20 trials, lies relatively close to the normalized visual cue reliability in all cases (Figure 8B). There is again a finite offset between normalized *w*_*v*_ and the normalized cue reliability, and additional trials do not reduce or eliminate this offset.

Overall, normalized *w*_*a*_ is relatively smaller than normalized *w*_*v*_ when the difference in noise levels between the auditory cue and visual cue is below +15 dB. This does not support the reliability-based cue weighting scheme because the relative reliability of the visual directional cue is set to be smaller than that of the auditory directional cue, which implies that normalized *w*_*v*_ should be lower than normalized *w*_*a*_. This is because the learned synaptic weight *w*_*a*_ is smaller than the learned synaptic weight *w*_*v*_ as described earlier, when the difference in noise levels between the auditory cue and visual cue is below +15 dB. When the difference in noise levels between the auditory cue and visual cue is above +15 dB, normalized *w*_*a*_ is relatively larger than normalized *w*_*v*_. As the relative noise level of the auditory directional cue decreases, i.e., as its relative reliability increases, its normalized synaptic weight *w*_*a*_ increases as well. Conversely, normalized *w*_*v*_ decreases when the relative noise level in the visual cue increases, i.e., its relative reliability decreases. This indicates that the relative synaptic weights follow changes in relative cue reliability, allowing for dynamic re-weighting of synaptic weights.

**Figure 9:**
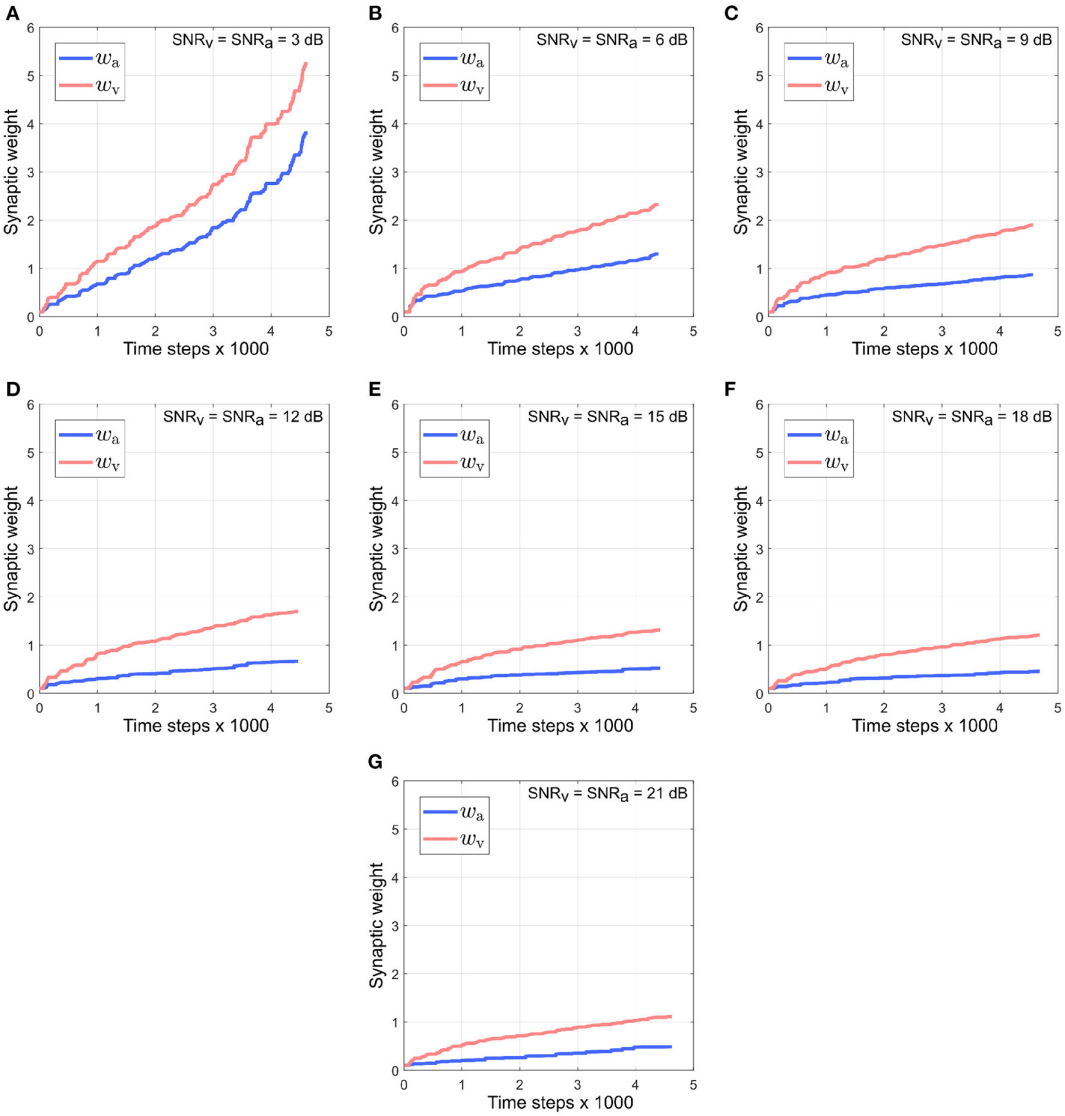
Evolution of synaptic weights *w*_a_ and *w*_v_ through the course of the trials. The SNR for both the visual and the auditory directional cues, SNR_v_ and SNR_a_, respectively, are kept identical and varied simultaneously in the range [3–21] dB **(A–G)**, in steps of 3 dB.

Figure 9 depicts the evolution of synaptic weights over the course of the trials when the noise in both modalities is kept identical and varied simultaneously in the range [3–21] dB. Under the reliability-based cue weighting scheme, the synaptic weights should reflect the relative cue reliabilities. This implies that when cues from different modalities have identical noise levels, their corresponding synaptic weights should also be identical. However, the synaptic weight of the visual cue rises quickly above that of the auditory cue. This can be explained by the dynamics of the auditory cue being larger than that of the visual cue due to the intermittent nature of the auditory cue. The intermittency results in relatively large fluctuations in the auditory cue relative to the visual cue, which is not intermittent and thus exhibits relatively small fluctuations. The synaptic weights *w*_*v*_ and *w*_*a*_ are, respectively, dependent on the dynamics of the auditory cue as encoded in its time derivative $\frac{dx_{a}}{dt}$ and the dynamics of the visual cue as encoded in its time derivative $\frac{dx_{v}}{dt}$. Since $\frac{dx_{a}}{dt}$ is greater than $\frac{dx_{v}}{dt}$, *w*_*v*_ is updated by a relatively larger amount, resulting in it rising above *w*_*a*_.

**Figure 10:**
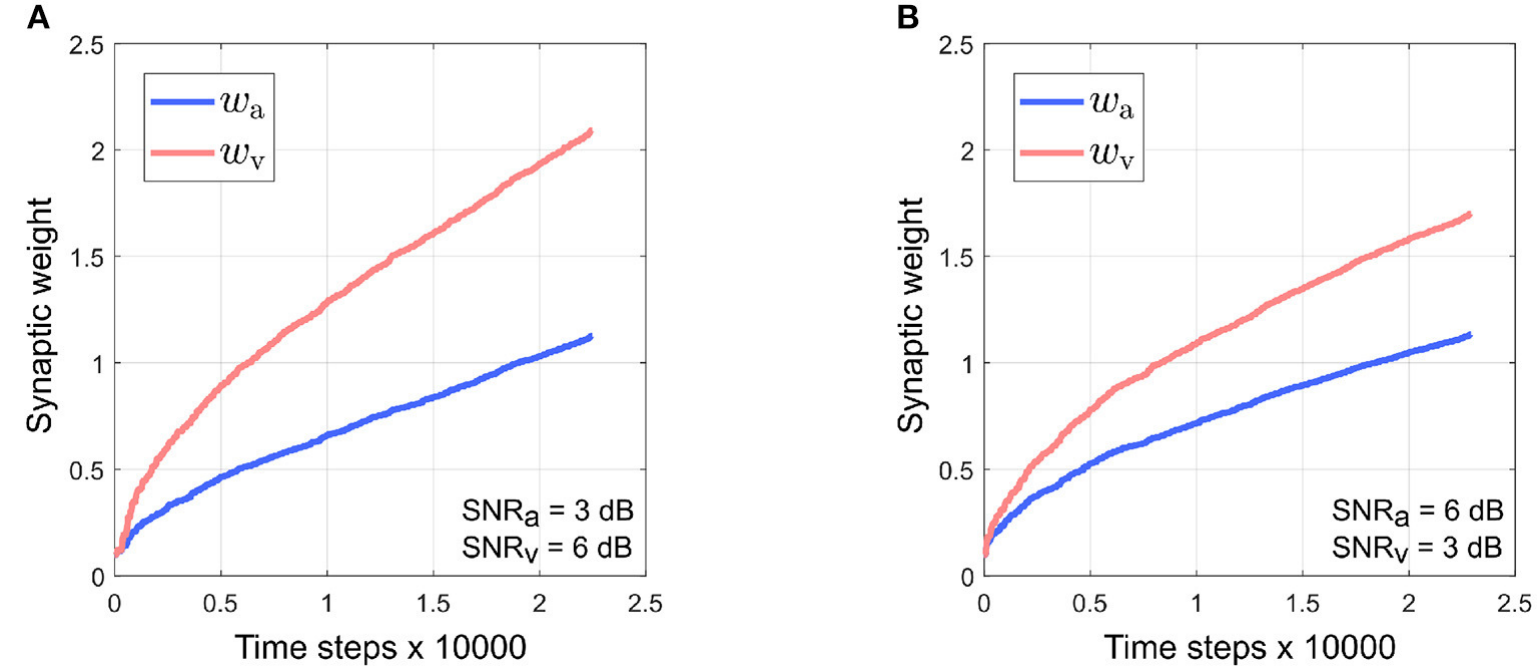
Stable evolution of synaptic weights *w*_a_ and *w*_v_ at low SNR levels over 100 trials. **(A)** SNR_a_ = 3 dB, SNR_v_ = 6 dB. **(B)** SNR_v_ = 3 dB, SNR_a_ = 6 dB. The learning rate is lowered to 0.01 as high learning rates can cause the robotic agent to exhibit large, undesirable oscillations in movement.

It can be observed that the absolute synaptic weights appear not to stabilize at low SNR levels, namely when SNR_a_ = 3 dB, SNR_v_ = 6 dB (Figure 5), SNR_v_ = 3 dB, SNR_a_ = 6 dB (Figure 7) and SNR_a_ = SNR_v_ = 3 dB (Figure 9). This is because 20 trials are insufficient to stabilize the weights. At low SNR levels, cue dynamics are relatively stronger, which results in greater weight updates. This also implies that more trials are required to stabilize the weights. This is evident in Figure 10 which depicts the evolution of the synaptic weights at low SNR levels over 100 trials.

### 3.1. Toward maximum-likelihood estimation in cue integration

**Figure 11:**
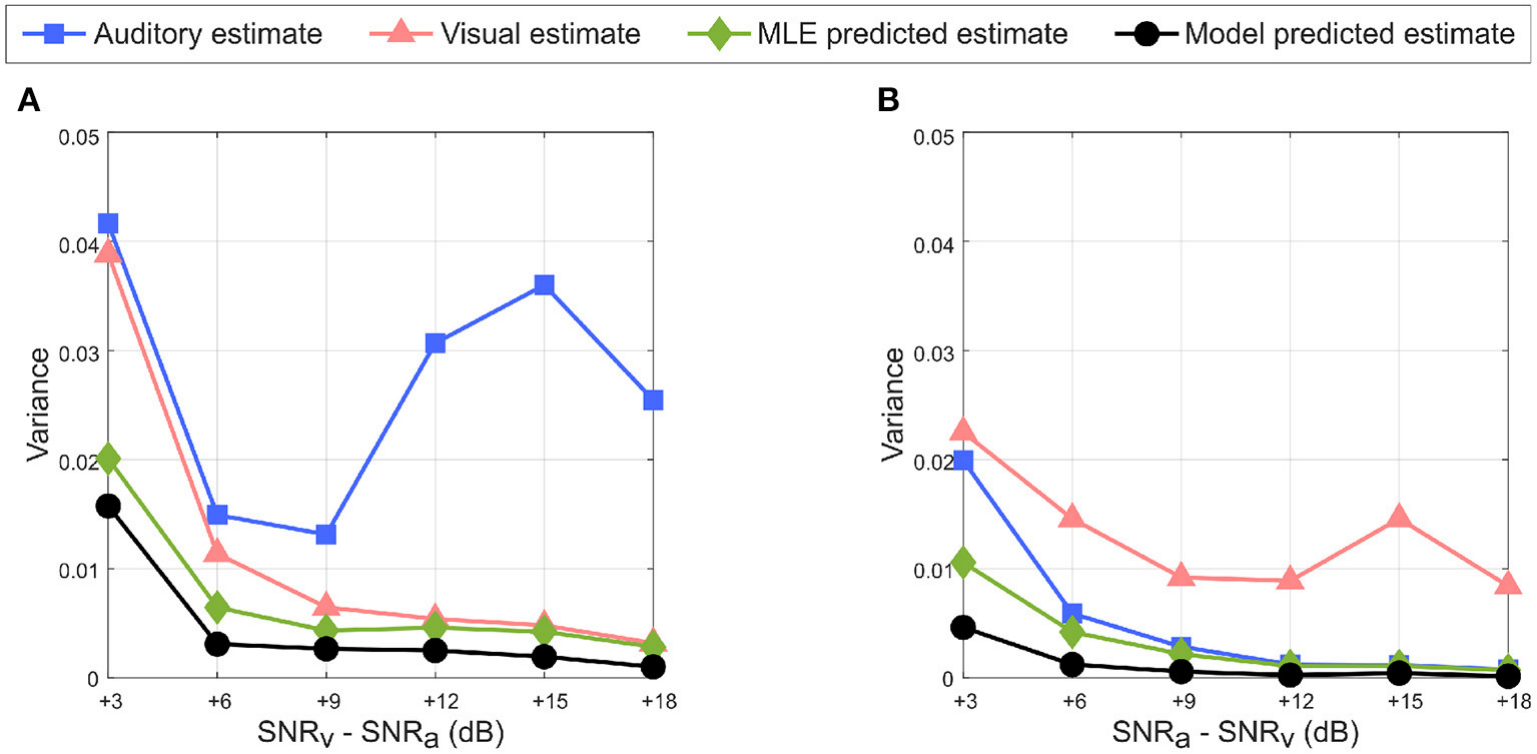
Reduction in uncertainty in multisensory response predicted by the model (in black with circular markers) with respect to the auditory estimate (in blue with square markers), visual estimate (in red with triangular markers) and MLE predicted estimate (in green with diamond markers). **(A)** SNR_v_ > SNR_a_. **(B)** SNR_a_ > SNR_v_. The multisensory responses of the proposed model have lower variance than either unisensory estimate, and follow the variance of the MLE predicted multisensory response.

An important requirement of the MLE hypothesis is that uncertainty in the combined multisensory estimate must be lower than that in unisensory estimate. This can be observed in Figure 11A when the auditory cue noise is kept fixed and visual cue noise is varied as well as in Figure 11B when the visual cue noise is kept fixed and auditory cue noise is varied. Since the learned unisensory weights for both cues reflect the relative cue reliabilities as illustrated in Figures 6, 8, the weighted summation of the auditory and visual cues results in an overall reduction in variance and hence in uncertainty in the multisensory cue combination. Due to the randomness introduced in MATLAB's noise generator (discussed at the end of Section 2.3), in Figure 11A the variance of the auditory cue estimate increases for SNR_v_> 9 dB, rather than decrease monotonically with increasing SNR. For the same reason the variance of the visual cue estimate for SNR_v_−SNR_a_ = 3 dB in Figure 11A is greater than that for SNR_a_−SNR_v_ = 3 dB in Figure 11B, even though SNR_v_ in the former case (6 dB) is greater than that in the latter case (3 dB).

To evaluate the performance of the proposed computational model in estimating relative target location in the tracking task, I compared it to the MLE model as the control condition using a simple linear regression model for comparison. Data (average relative target location predicted by the computational model) was generated from 50 randomized trials conducted in similar manner to those described in Section 2.6, except that the learning rules were disabled and the auditory and visual synaptic weights were set to fixed values. In each trial, target tracking was performed using the proposed computational model as well as a MLE model as control condition. For the proposed computational model, the synaptic weights for either modality were set to their corresponding normalized values learned after the 20 trials conducted earlier (Figures 5, 7, 9). For the MLE model, the synaptic weights for either modality were set to their corresponding normalized cue reliabilities calculated as the inverse of the variance in the corresponding cues. The SNRs for both modalities were randomly assigned before each trial, satisfying one of two possible conditions–(a) both modalities are randomly assigned identical SNRs (set as either 3, 9, 12, 15, 18, or 21 dB) or (b) a randomly chosen modality is randomly assigned a SNR (either 3, 9, 12, 15, 18, or 21 dB) while the other modality is assigned a SNR of 3 dB.

The regression analysis was performed in MATLAB R2021b (Mathworks Inc.) *via* the available Curve Fitting toolbox. The linear regression model used was a 1st-order polynomial of the form *f*(*x*) = *p*1*x*+*p*2, where *p*1 and *p*2 are the regression coefficients. The regression model used the bisquare weighting method implemented in the Curve Fitting toolbox to fit the regression line. This method attempts to minimize a weighted sum of squares over all the data points, where each data point is weighted according to how far it lies from the fitted line. Data points near the fitted line are assigned maximum weight, while data points farther from the fitted line are assigned a correspondingly smaller weight. Data points lying farther from the fitted line than as expected by random chance are assigned zero weight. This allows the bisquare method to find a curve that fits the bulk of the data points *via* the least-squares approach, as well as to minimize the effect of outliers.

**Figure 12:**
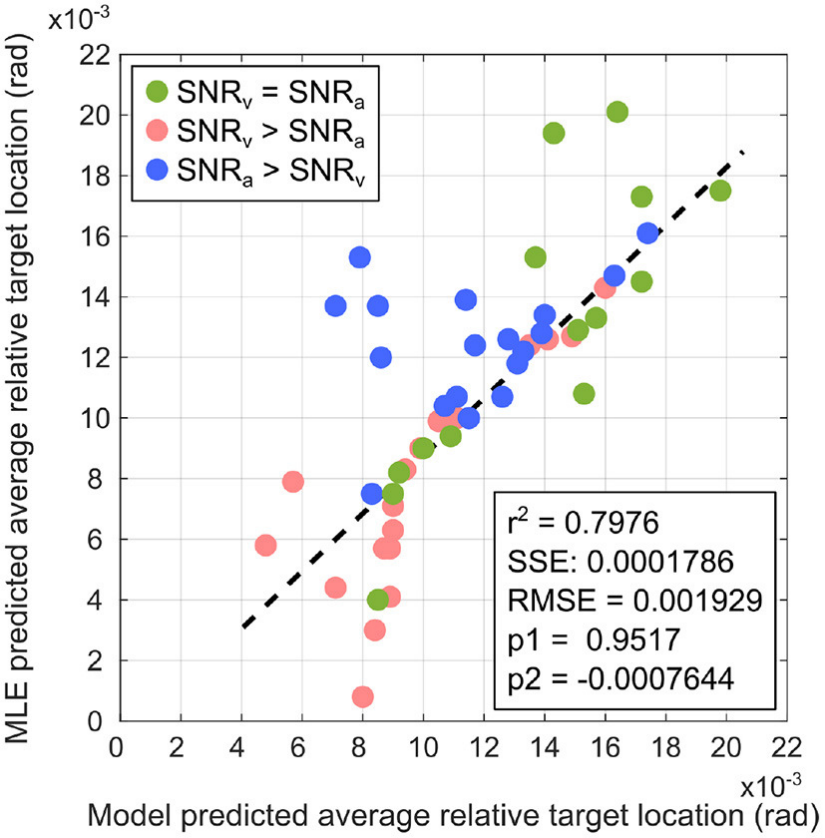
Comparison of the proposed computational model with the MLE model *via* linear regression for SNR_v_ = SNR_a_ (green markers), SNR_v_>SNR_a_ (red markers), and SNR_a_>SNR_v_ (blue markers). The linear regression model is a 1st-order polynomial of the form *f*(*x*) = *p*1*x*+*p*2, where *p*1 and *p*2 are the regression coefficients. Relevant regression measures (coefficient of determination *r*^2^), sum of squared error SSE, root mean square error RMSE) indicate that the proposed computational model is a relatively good fit to the MLE model.

Figure 12 depicts the linear regression fit of the average target location predicted by the proposed computational model with respect to that predicted by the MLE model. The analysis generates a coefficient of determination *r*^2^ = 0.7976. Fitting a regression line *via* simple linear regression is equivalent to determining the degree of correlation between two sets of data points, and one can determine the correlation coefficient as $\sqrt{r^{2}}$ = *r* = 0.8931. This relatively large positive correlation between the proposed computational model and the MLE model suggests that the proposed computational model approximates MLE-like computation. This suggests that the crossmodal synaptic plasticity rules implemented in the proposed computational model enable learning of a multisensory cue integration model that could act as a precursor to learning reliability-weighted cue integration according to the MLE model.

As discussed earlier in Section 2.3, changes in cue SNR may not directly correspond to equivalent changes in cue variances and thus reliabilities as evident in Figure 11. I used two-sample, one-tailed, unpaired t-tests to determine whether a multisensory model's estimate of target location was significantly higher (i.e., implying a lower localization error) than an estimate from a single modality cue. Separate t-tests were performed for the proposed model as well as for the MLE model, to compare each of their estimates to both the auditory cue only and the visual cue only estimates, resulting in four separate *t*-tests. The null hypothesis in all cases was that there is no difference between a multisensory model's estimate of target location and an estimate from a single modality cue. I used MATLAB's built-in function *ttest2* (*x, y*, “*VarType*,” “*unequal*,” “*Tail*,” “*right”*) implemented in the MATLAB Statistics and Machine Learning toolbox, to perform the four *t*-tests for each of the three conditions—(a) SNR_v_>SNR_a_, (b) SNR_a_>SNR_v_, and (c) SNR_v_ = SNR_a_. The argument “*VarType*,” “*unequal”* implies the assumption that the variances of *x* and *y* are unequal. The argument “*Tail*,” “*right”* tests against the alternative hypothesis that the population mean of *x* (set as either the proposed model estimate or the MLE model estimate) is greater than the population mean of *y* (set as either auditory-only estimate or visual-only estimate). The results of the statistical analyses (Table 1) indicate that in all cases, the null hypothesis that there is no difference between a multisensory model's estimate of target location and an estimate from a single modality cue is rejected at the 5% statistical significance level. This suggests that both the proposed model and the MLE model estimates are significantly better than single modality cue estimates.

**Table 1:** Results of two-sample, one-tailed, unpaired *t*-tests for statistical significance between multisensory model estimates (both the proposed model estimates as well as the MLE model estimates) and single modality cue estimates (both auditory-only and visual-only cue estimates).

		**Auditory-only estimates**	**Visual-only estimates**
SNR_v_>SNR_a_	Proposed model estimates	t = 15.2037	t = 2.3771
		df = 20.4972	df = 33.5924
		*p* = 6.2337 ×10^−13^	*p* = 0.0116
	MLE model estimates	*t* = 11.4085	*t* = 10.176
		df = 17.1074	df = 17.3598
		*p* = 1.0114 ×10^−9^	*p* = 4.8797 ×10^−9^
SNR_a_>SNR_v_	Proposed model estimates	*t* = 11.6588	*t* = 10.6143
		df = 17.0302	df = 17.4578
		*p* = 7.6539 ×10^−10^	*p* = 2.4371 ×10^−9^
	MLE model estimates	*t* = 4.0633	*t* = 6.5891
		df = 33.96	df = 26.0925
		*p* = 1.3522 ×10^−4^	*p* = 2.6933 ×10^−7^
SNR_v_ = SNR_a_	Proposed model estimates	*t* = 8.0346	*t* = 7.2253
		df = 13.0197	df = 13.4682
		*p* = 1.0559 ×10^−6^	*p* = 2.738 ×10^−6^
	MLE model estimates	*t* = 5.9809	*t* = 4.893
		df = 24.4811	*df* = 25.2756
		*p* = 1.6476 ×10^−6^	*p* = 2.3913 ×10^−5^

*In all cases, the null hypothesis that there is no difference between a multisensory model's estimate of target location and an estimate from a single modality cue is rejected at the 5% statistical significance level. This suggests that both the proposed model and the MLE model estimates are significantly better than single modality cue estimates*.

## 4. Conclusions

I hypothesized that experience dependent crossmodal synaptic plasticity may be a plausible mechanism underlying development of multisensory cue integration. I presented a computational model for learning multisensory cue integration that utilized symmetric crossmodal learning across modalities to learn synaptic weights that reflect relative cue reliabilities. The model assumed no prior knowledge about the sensory cues and implemented multisensory cue integration *via* a naive reliability-based cue weighting scheme. The model was embodied in a simulated robotic agent tasked with localizing a randomly moving audio-visual target by integrating cues encoding its spatial location, extracted from of auditory and visual sensory modalities. The embodiment of the model in the task environment generated rich sensorimotor experiences that drove synaptic weight updates. Simulation trials demonstrated that the model was able to capture stimulus statistics and learn modality-specific synaptic weights that were proportional to the relative reliabilities of the auditory and visual cues.

One interesting observation that emerged from the simulations is that the synaptic weight for the visual cue was relatively larger than that of the auditory cue, even when the latter was more reliable than the former. Only when the auditory cue was significantly cleaner as compared to the visual cue (conversely, when the visual cue was significantly degraded as compared to the auditory cue), the auditory cue weight rose marginally above the visual cue weight. This was a result of the differences in dynamics between the auditory and visual cues and the crossmodal influences implemented in the model as described earlier. It has been reported that audition could dominate vision when the spatial cues provided by vision were sufficiently degraded (Alais and Burr, 2004). The consensus in the scientific community is that the brain estimates the instantaneous precision of individual sensory cues (van Beers et al., 1999; Ernst and Banks, 2002; Roach et al., 2006; Van Dam et al., 2014). The brain may use these estimates when integrating the individual cues to form a unified percept.

It must be noted that in all trials the learned synaptic weights did not exactly match the relative cue reliabilities. This is not consistent with MLE and implies that cue integration implemented by the proposed model does not follow an “optimal” weighted cue summation process where the weights directly and accurately encode cue reliabilities. Recent psychophysical data from localization trials in humans suggests that multisensory cue integration does not follow a precision weighted summation process, suggesting that multisensory cue integration is sub-optimal (Arnold et al., 2019). Furthermore, recent psychophysical data from large-scale human audio-visual localization experiments suggests that audio-visual spatial cues are not weighted exactly in proportion to their reliabilities (Meijer et al., 2019). This suggests that additional information about the sensory modalities must be taken into account to get the cue weights to exactly match relative cue reliabilities and achieve “optimal” cue integration as in the MLE model. This additional information could in principle be prior knowledge about the statistics of the cues, reflecting a priori beliefs about the causal nature of multisensory events, essentially implementing a statistically optimal Bayesian estimator, i.e., an “ideal” observer model.

Finally, the computational model proposed in this study does not account for causal inference, which is an important precursor to multisensory cue integration. Causal inference involves determining whether multimodal sensory cues arise from the same source, and is necessary for the brain to decide whether the cues should be integrated or segregated. While a number of behavioral and computational studies on causal inference have been conducted, the neural mechanisms underlying causal inference have yet to be fully investigated (French and DeAngelis, 2020).

## Data availability statement

The original contributions presented in the study are included in the article/Supplementary material, further inquiries can be directed to the corresponding authors.

## Author contributions

DS is solely responsible for experimentation, data analysis, and preparation of manuscript.

## Conflict of interest

The author declares that the research was conducted in the absence of any commercial or financial relationships that could be construed as a potential conflict of interest.

## Publisher's note

All claims expressed in this article are solely those of the authors and do not necessarily represent those of their affiliated organizations, or those of the publisher, the editors and the reviewers. Any product that may be evaluated in this article, or claim that may be made by its manufacturer, is not guaranteed or endorsed by the publisher.
